# Developmental Ethanol Exposure Leads to Dysregulation of Lipid Metabolism and Oxidative Stress in *Drosophila*

**DOI:** 10.1534/g3.114.015040

**Published:** 2014-11-11

**Authors:** Theresa Logan-Garbisch, Anthony Bortolazzo, Peter Luu, Audrey Ford, David Do, Payam Khodabakhshi, Rachael L. French

**Affiliations:** Department of Biological Sciences, San José State University, 1 Washington Square, San José, California 95192-0100

**Keywords:** fetal alcohol syndrome, reactive oxygen species, lipid accumulation, withered, carnitine transporter

## Abstract

Ethanol exposure during development causes an array of developmental abnormalities, both physiological and behavioral. In mammals, these abnormalities are collectively known as fetal alcohol effects (FAE) or fetal alcohol spectrum disorder (FASD). We have established a *Drosophila melanogaster* model of FASD and have previously shown that developmental ethanol exposure in flies leads to reduced expression of insulin-like peptides (dILPs) and their receptor. In this work, we link that observation to dysregulation of fatty acid metabolism and lipid accumulation. Further, we show that developmental ethanol exposure in *Drosophila* causes oxidative stress, that this stress is a primary cause of the developmental lethality and delay associated with ethanol exposure, and, finally, that one of the mechanisms by which ethanol increases oxidative stress is through abnormal fatty acid metabolism. These data suggest a previously uncharacterized mechanism by which ethanol causes the symptoms associated with FASD.

Developmental exposure to ethanol causes a constellation of developmental and neurobehavioral problems in organisms from humans to *Drosophila*. These include slow growth, developmental delays (both physical and intellectual), reduced brain size, low birth weight, and a variety of behavioral and intellectual disabilities, including learning and memory deficits, sleep difficulties, impulse control problems, and reduced executive function (Hanson *et al.* 1976; Streissguth *et al.* 1980, 2004; Colangelo and Jones 1982; Kodituwakku 2007). In humans, these symptoms are referred to collectively as “fetal alcohol syndrome” or “fetal alcohol spectrum disorders” (FASD), reflecting the fact that the results of fetal ethanol exposure can vary substantially from individual to individual. In this article, we use the term “developmental alcohol exposure” (DAE) because it encompasses mammalian and invertebrate development.

The variation in symptoms arising from DAE likely reflects a number of factors, including dose, timing, and duration of exposure (Guerri *et al.* 2009; Nunez *et al.* 2011). In addition, in twin studies of FASD in humans, greater concordance of phenotype was observed in identical twin pairs compared with fraternal twin pairs, indicating either a genetic effect on the susceptibility to the negative effects of DAE or effects of placental development on the amount of ethanol fetuses are exposed to (Warren and Li 2005).

Ethanol affects the activity or expression of numerous molecules and pathways, including membrane lipids, a variety of transcription factors, the epidermal growth factor/mitogen-activated protein kinase (EGFR/MAPK) signaling pathway, 5-HT, and dopamine receptors, and insulin-like growth factors (IGFs) and their receptors (Harris *et al.* 2008; Corl *et al.* 2009; McClure *et al.* 2011) (our unpublished data). Although a few of these (MAPK and IGF signaling in particular) have been shown to be important for ethanol’s developmental effects in animal models, identification of the relevant targets and elucidation of ethanol’s mechanism(s) of action have been largely elusive. This is almost certainly due to the breadth of phenotypes associated with DAE, combined with the wide variety of potential targets.

The combination of the widespread socioeconomic and health effects of DAE and the relative ineffectiveness of public awareness campaigns makes an understanding of the molecular mechanisms of harm caused by developmental ethanol exposure critical for efforts in prevention and mitigation. We have developed a model of DAE in the genetic model organism *Drosophila melanogaster*. We have previously established that many of the phenotypes resulting from DAE are similar between flies and mammals, and that at least one of the major molecular targets (IGF signaling) is conserved (McClure *et al.* 2011).

There are data indicating a role for oxidative stress in phenotypes resulting from DAE. In general, amelioration of oxidative stress tends to improve outcomes in animal models (Mitchell *et al.* 1999; Heaton *et al.* 2000; Marino *et al.* 2004; Peng *et al.* 2005; Wentzel and Eriksson 2006; Wentzel *et al.* 2006; Shirpoor *et al.* 2009). These data are indirect in that there is substantial overlap between the molecular pathways that detoxify ethanol and those that reduce free radicals and correct oxidative damage (Lieber 1997; Wu and Cederbaum 2003).

In addition, there is direct evidence that DAE leads to increased oxidative stress in several models. Ethanol exposure increases the production of reactive oxygen species (ROS) in fetal liver tissue (Chen *et al.* 1997) and brains (Heaton *et al.* 2002). Ramachandran *et al.* (2003) observed increased apoptosis in cultured rat neurons after ethanol exposure in combination with significant increases in ROS. Studies directly connecting oxidative damage to ethanol-induced developmental phenotypes are elusive; however, it seems likely that at least some of the deleterious effects of DAE are due to oxidative stress.

In adult *Drosophila*, reduced insulin-like signaling peptide (dILP) levels lead to an increase in circulating triglycerides as well as increased lipid storage in the fat body (Hwangbo *et al.* 2004; Broughton *et al.* 2005; Pospisilik *et al.* 2010). These obese animals are sensitive to some cellular stresses, including heat and cold, but are resistant to oxidative stress and have an increased lifespan. These observations have led to the hypothesis that reduced insulin signaling leads to an increase in lifespan by conferring resistance to the deleterious effects of ROS.

None of the studies described above examines the effects of developmental alteration of insulin signaling and the resulting increased storage of fat. Furthermore, it is not known whether the lipid accumulation in these studies was specific to the fat body or had spread to nonadipose tissues as well. Lipid accumulation in nonadipose tissues is known to be toxic due to caspase-mediated apoptosis triggered by a combination of ceramide accumulation and high increased production of ROS due to high levels of palmitoyl-CoA (Listenberger *et al.* 2001, 2003). In such cases, triglyceride accumulation promoted by the presence of unsaturated fatty acids is a protective mechanism against cellular toxicity. Thus, triglyceride accumulation is a biomarker for fatty-acid–induced cellular toxicity.

Here, we present data showing that the developmental delay and lethality associated with DAE are largely due to oxidative stress. We demonstrate that ethanol causes oxidative stress through reduced antioxidant gene expression and provide evidence that disruption of lipid metabolism contributes to the toxic effects of DAE. The latter mechanism has not previously been implicated in FASD in mammals and may represent a potential new approach to the understanding of FASD and its treatment. Finally, our data suggest that the relationship between insulin signaling and oxidative stress resistance in development may be more complicated than previously anticipated.

## Materials and Methods

### Fly stocks and genetics

Fly stocks were maintained at 25° on standard corn meal/molasses medium. With the exception of *Cat^n1^*, *whd^1^*, and RNAi strains from the Transgenic RNAi Project (TRiP), all mutant alleles and transgenes were introgressed for five generations into our standard laboratory background (*w^1118^*; Wild-Type Berlin (*w*; *WTB*)). Fly strains were obtained from the Bloomington *Drosophila* Stock Center (Bloomington, Indiana). Strains used were: *w^1118^*; *Pdk1^EP3091^*, *w^1118^*; *daGAL4*, *w^1118^*; *UAS-P{Cat.A}2*, *w^1118^*; *UAS-P{TRiP.GL01541}attP40*, *w^1118^*; *w^1118^*; *GS^EP1322^*, *w^1118^*; Pxd^EY15388^, ry^506^, *whd^1^*, *w^1118^*; *whd^KG01596^*, *w^1118^*; *whd^KG01596^*, *w^1118^*; *Cat^e01301^*, *Cat^n1^/TM3*, *Sb^1^ Ser^1^*, and *w^1118^*; *uro^f04888^*.

For the *Catalase* transgenic expression experiments, *daughterless-GAL4* (*daGAL4*) virgin females were crossed to *UAS-Cat^RNAi^* or *UAS-Cat^+^* males. Controls for transgene insertion effects were generated by crossing *w*; *WTB* virgin females to males from the corresponding UAS line, as well as *daGAL4* virgin females to *w*; *WTB* males.

### Survival and development time assays

Egg collections were taken for 16–20 hr on Petri dishes containing standard fly food. One hundred eggs were then transferred to vials containing either ethanol-containing food, peroxide-containing food, or control food, and, for ethanol exposure experiments, placed in a 3–8% ethanol bath (experimental conditions; ethanol concentration matches the concentration in the food) or water bath (control conditions). The ethanol bath ensures that developing animals are exposed to ethanol during their entire development, which continues for another 10–16 d. The number of newly eclosed adult flies was counted daily between 9 and 21 d after egg laying, and these data were used to generate cumulative eclosion rate plots, a direct measurement of egg-to-adult survival, and the time to 50% of total eclosion. Time to 50% eclosion was calculated by linear interpolation.

### Starvation assay

*w*; *WTB* flies were reared in control medium or food containing 7% ethanol. After eclosion, 0- to 2-d-old adult flies were transferred to vials containing either standard corn meal/molasses medium (control conditions) or 1% agarose in water (starvation conditions). Flies were transferred to new vials daily for 6 d (the time at which all starved flies had died), and the surviving flies were counted.

### Quantitative RT-PCR and microarray analysis

For quantitative reverse-transcriptase–mediated PCR (qRT-PCR) and microarray analysis, third instar larvae were snap-frozen on dry ice. Total RNA was extracted using Trizol reagent (Life Technologies, Carlsbad, CA) according to the manufacturer’s instructions, resuspended in RNase-free water, and stored at −80° until use. For qRT-PCR, 2 μg of total RNA was reverse-transcribed using the High-Capacity RNA-to-cDNA Kit (Applied Biosystems, Carlsbad, CA) according to the manufacturer’s instructions. cDNA was analyzed by quantitative real-time PCR using the Applied Biosystems 7300 Real-Time PCR System (Applied Biosystems). The *rp49* transcript levels were used as an endogenous normalization control for RNA samples, and relative mRNA abundance was calculated using the comparative ΔCt method (Schmittgen and Livak 2008). Each sample was analyzed in triplicate. As negative controls, we used both no-template and DNAse-treated nonreverse-transcribed mRNA samples; no significant amplification was observed in these samples.

For microarray analysis, total larval RNA was hybridized to Affymetrix *Drosophila* 2.0 oligonucleotide microarray chips at the Partners HealthCare Center for Personalized Genetic Medicine Microarray Facility (Harvard University). Gene expression changes were quantified using a linear models approach (Smyth 2004) and initially ranked by the magnitude of the difference in expression change on ethanol compared with control food.

### Overrepresentation analysis

We used the GO Elite software package (Gladstone Institutes) according to the manufacturer’s directions to identify Gene Ontology terms that were overrepresented in the set of transcripts whose expression was altered by at least four-fold in ethanol-reared larvae relative to the entire dataset. The 1666 transcripts altered by four-fold or more were the input, or “numerator”, whereas the total set of 18,952 transcripts represented on the array was the “denominator.” The software then uses two statistics, the Z score statistic and Fisher’s exact test, to assess overrepresentation in the input of certain gene ontology terms.

### Lipid droplet staining and imaging

Larvae were dissected in PBS and fixed in 4% paraformaldehyde in PBS for 30 min at room temperature. Tissues were then rinsed twice with 1× PBS, then incubated for 30 min in a 1:1000 of 0.05% Nile Red (Sigma, St. Louis, MO); 2 ng/ml DAPI was used to stain nuclei. Stained samples were mounted in 75% glycerol for microscopy analysis. All images were collected on a Zeiss LSM 700 confocal microscope at 200× magnification.

### Primer sequences

*Cat*: Left primer: 5′-GAATTCTCGACGCAGTCACA-3′. Right primer: 5′-CTGCAGCAGGATAGGTCCTC-3′
*GS*: Left primer: 5′-AGTTCACGGCCAATCTGTTC-3′. Right primer: 5′-ATCCTGACCACGATCCTCAC-3′
*Pxd-RA* and –*RC*: Left primer: 5′-TAAGCCCTCGATTTTTGTGG-3′. Right primer: 5′-GCCAAGAGCAGAAGATCTCG-3′
*Pxd-RB*: Left primer: 5′-CGAACTGCCATTTAGCTGGT-3′. Right primer: 5′-TCACATATGGAGCTGATCCTTG-3′
*uro*: Left primer: 5′-CGACTTCAGCTCCATTGACA-3′. Right primer: 5′-AATCCACCACGGTGCTAAAG-3′
*Sod*: Left primer: 5′-TTGCCATACGGATTGAAGTG-3′. Right primer: 5′-CGAACAGGAGGTGAGAATCC-3′
*Cyp4e3*: Left primer: 5′-ATTGCTTGGGATTGGTCATC-3′. Right primer: 5′-CTGCCACTGATCACATTGGT-3′

## Results

### Mutation of *Pdk1* alters the developmental response to ethanol

**Figure 1:**
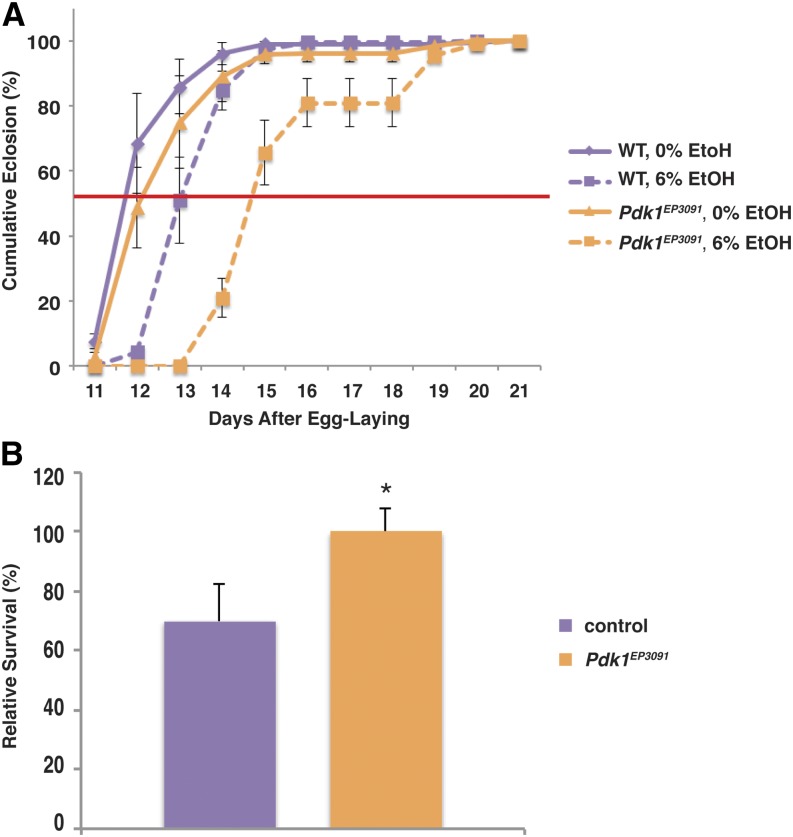
Mutation of Pdk1 alters the developmental response to ethanol. (A) *Pdk1^EP3091^* mutant flies are sensitive to ethanol-induced developmental delay. The red line indicates 50% eclosion. Times to 50% eclosion were: WT 0% EtOH, 11.85 ± 0.26 d; WT 6% EtOH, 13.05 ± 0.24 d (not significant, one-way ANOVA with Tukey HSD post hoc analysis); *Pdk1^EP3091^* 0% EtOH, 12.76 ± 0.51 d (not significant, one-way ANOVA with Tukey HSD posthoc analysis); and *Pdk1^EP3091^* 6% EtOH, 14.75 d ± 0.25 d (*P* < 0.01 *vs.* both 0% controls, one-way ANOVA with Tukey HSD post hoc analysis). Data are normalized to the total number of flies that eclosed for that treatment condition and genotype. (B) *Pdk1^EP3091^* mutant flies are resistant to ethanol-induced developmental lethality. Survival has been normalized to age- and genotype-matched controls reared in ethanol-free food. N = 4; *P* = 0.044, Student’s t-test.

Ethanol exposure during larval development leads to reduced survival and increased development time relative to unexposed controls, and these phenotypes are, at least in part, due to reduced insulin signaling (McClure *et al.* 2011). While investigating the functions of the pathways downstream of the insulin receptor in these phenotypes, we found that while mutation of *Pdk1* results in the expected enhancement of ethanol’s effects on development time, it has the opposite effect on survival (Figure 1). Control flies in ethanol-free food reach median eclosion 11.85 d after egg laying, and addition of 6% ethanol to the growth medium increases this time to 13.05 d, which is an increase of 1.2 d. Flies homozygous for *Pdk1^EP3091^* reach median eclosion in control food at 12.76 d, whereas *Pdk1^EP3091^* flies reared in 6% ethanol take an additional 2 d (median eclosion = 14.75 d) (Figure 1A and Table 1).

**Table 1:** Quantitation of development time for data in Figure 2

Condition	Time to Median Eclosion (d)	Difference (d)
control	11.67 ± 0.08	N/A
3% ethanol	11.75 ± 0.05	+0.08 (not significant)
7.4 mM H2O2	12.28 ± 0.08	+0.61 (*P* < 0.01)
8.8 mM H2O2	11.78 ± 0.06	+0.11 (not significant)
3% ethanol + 8.8 mM H2O2	12.57 ± 0.09	+0.9 (*P* < 0.01, all comparisons)
10.3 mM H2O2	13.15 ± 0.14	+1.48 (*P* < 0.01)
11.8 mM H2O2	13.33 ± 0.17	+1.66 (*P* < 0.01)
3% ethanol + 11.8 mM H2O2	14.48 ± 0.17	+2.81 (*P* < 0.01, all comparisons)

Data are presented as time to median eclosion ± SEM. “Difference” is time to median eclosion for the untreated control minus time to median eclosion for the indicated experimental treatment. For single treatments, *P* values indicate a comparison between the untreated control and the treatment. For combinations of ethanol and peroxide, comparisons were made between untreated controls as well as each of the single treatment controls. One-way ANOVA with Tukey HSD post-hoc analysis.

When we examined the survival of the flies, however, we found that the *Pdk1^EP3091^* mutation resulted in complete suppression of the deleterious effects of ethanol on larval survival. On average, 70% of control flies survived to eclosion when reared in 6% ethanol, whereas the survival of *Pdk1^EP3091^* mutant flies was the same in ethanol-containing media and control food (Figure 1B). In all figures, we present survival as the percentage of flies of a given genotype that survive when reared in ethanol relative to flies of the same genotype reared in control food. This allows us to separate mutant strains that are unhealthy for reasons unrelated to ethanol exposure from those where the mutant is specifically sensitive to ethanol exposure. In this case, *Pdk1^EP3091^* flies show only a small but statistically insignificant effect on survival (84% compared with wild-type flies in control food, *P* = 0.19, Student’s t test), which, as indicated above, is unchanged by ethanol exposure.

This result has two implications. First, the growth and survival phenotypes are mechanistically separable, as the same mutation results in opposing phenotypes. This is not particularly surprising, because our previous work demonstrated that the critical developmental periods for ethanol-induced lethality and developmental delay were separable (McClure *et al.* 2011). Second, given that *Pdk1^EP3091^* affects both phenotypes, but in different ways, it would appear that the effects of insulin signaling through the phosphoinositide 3-kinase (PI3K) and phosphoinositide-dependent kinase (Pdk) pathway in response to ethanol is not as straightforward as a simple downregulation of *dilp* and *InR* expression as a result of ethanol exposure.

### Oxidative stress phenocopies the effects of ethanol on growth and development

In addition to their well-characterized roles in growth and development, IGFs are implicated in a variety of other biological pathways, including the regulation of age-related phenotypes resulting from oxidative stress. Specifically, two alleles of *Pdk1* have been shown to be resistant to oxidative stress and age-related locomotor impairment (Jones *et al.* 2009). Because ethanol is known to increase the production of ROS (Chen *et al.* 1997; Heaton *et al.* 2002; Ramachandran *et al.* 2003), we hypothesized that ethanol-induced larval mortality is, in large part, due to the increased production of ROS, and that mutation of *Pdk1* increases survival through increased resistance to oxidative stress.

**Figure 2:**
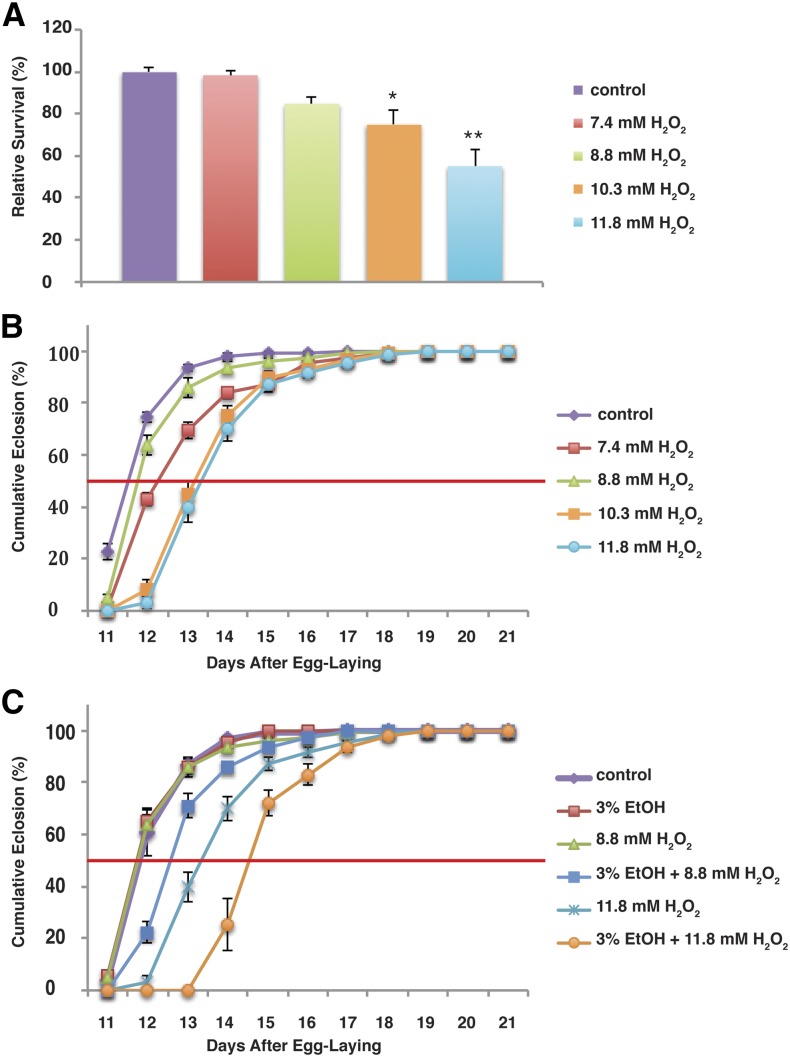
Oxidative stress phenocopies the effects of ethanol on survival and development time. Wild-type flies were reared on increasing concentrations of H_2_O_2_ and counted as they eclosed. (A and B) H_2_O_2_ reduces survival and increases development time in a dose-dependent fashion. (C) Ethanol and H_2_O_2_ have a synergistic effect on development time. Sub-threshold concentrations of ethanol and H_2_O_2_ have no effect when administered separately, but when combined they lead to a significant increase in development time. In addition, low concentrations of each drug have a nonadditive effect when administered together. In (A), data are normalized to the survival of flies on control (drug-free) food. In (B) and (C), data are normalized to the total number of flies that eclosed for that treatment condition. See Table 1 for quantitation of mean eclosion times. *P < 0.05. **P < 0.01.

To test this hypothesis, we first exposed larvae to increased oxidative stress by rearing them in hydrogen peroxide. We found that hydrogen peroxide results in a dose-responsive increase in both mortality and development time (Figures 2, A and B).

Development time and lethality are fairly nonspecific tests, so to determine whether ethanol and hydrogen peroxide are exerting their effects through the same targets, we exposed animals simultaneously to both drugs. The results of these experiments are shown in Figure 2C. We exposed flies to a combination treatment of 3% ethanol and 8.8 mM hydrogen peroxide. Neither treatment, independently, has any effect on development time, whereas 8.8 mM hydrogen peroxide has a small but insignificant effect on survival to eclosion (Figures 2, A–C and Table 1). However, when the treatments were combined, we saw a significant effect on development time. Flies receiving the combination treatment had a median eclosion of 12.57 d compared with 11.8 d for the separate treatments. In addition, 11.8 mM hydrogen peroxide alone increases development time by 1.66 d compared with controls; however, when we added 3% ethanol to the treatment, development time increased by another 1.2 d (Table 1). Thus, the effects of ethanol and hydrogen peroxide on development are nonadditive, and this synergistic effect strongly suggests that the molecular targets of the two drugs significantly overlap.

### Genetic manipulation of oxidative stress alters the developmental effects of ethanol

**Figure 3:**
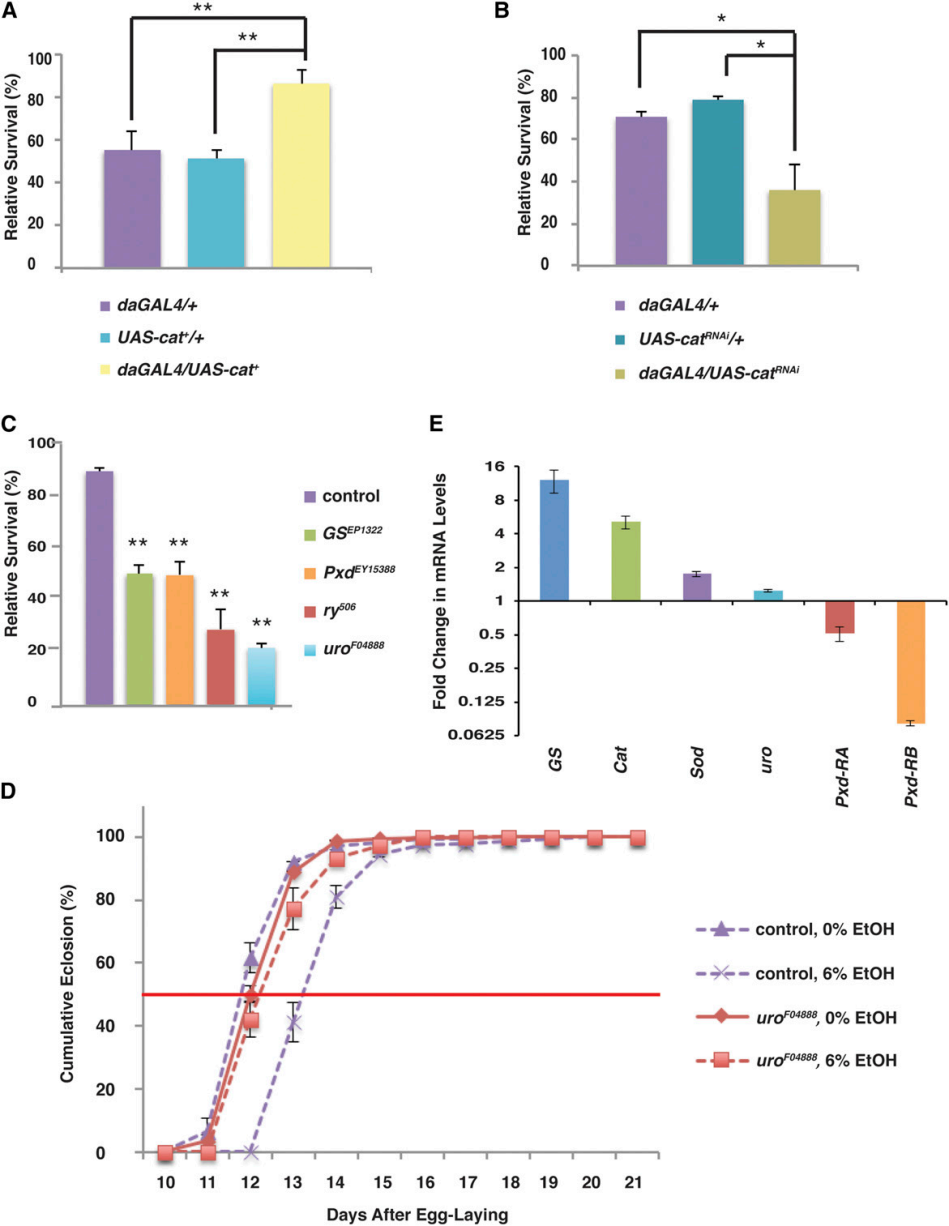
Developmental ethanol exposure causes increased oxidative stress and changes in antioxidant gene expression. (A) Upregulation of Cat is protective against ethanol-induced developmental mortality. N = 4. (B) Ubiquitous downregulation of *Catalase* increases ethanol-induced developmental mortality. N = 4. (C) Flies with mutations in antioxidant genes are sensitive to ethanol-induced developmental lethality. Survival has been normalized to age- and genotype-matched controls reared in ethanol-free food. N = 6. (D) Mutation of *uro* leads to resistance to ethanol-induced developmental delay. The development time of *uro* mutant flies is the same as development time in control food (N = 6). *P < 0.05 **P < 0.01 (one-way ANOVA with Tukey HSD post hoc analysis). (E) Antioxidant gene expression is altered in ethanol-reared larvae. qRT-PCR analysis shows that expressions of the antioxidant genes *GS*, *Cat*, and *Sod* are increased in ethanol-reared larvae, but *Pxd* expression is reduced. Levels of *uro* mRNA are not significantly changed.

We next specifically tested the role of oxidative stress in the deleterious effects of ethanol by using genetic methods to increase or decrease the ability of larvae to respond to reactive oxygen species. We first used the ubiquitously expressed *daGAL4* driver to drive expression of peroxisomal Catalase (*UAS-Cat^+^*). Flies overexpressing Cat in this fashion were strikingly resistant to ethanol exposure: 86% of *daGAL4/UAS-Cat^+^* flies survived to eclosion after exposure to ethanol during development, whereas only 51–55% of control flies survived (Figure 3A). Similarly, when we ubiquitously downregulated Cat through expression of a double-stranded RNA interference construct (*UAS-Cat^RNAi^*), we observed a dramatic reduction in survival: only 36% of *daGAL4/UAS-Cat^RNAi^* flies survived compared with 70–79% of control flies (Figure 3B). In addition, flies homozygous for the viable loss-of-function allele *Cat^e01301^* show a similar reduction in viability when reared in ethanol: only 38% of *Cat^e01301^* flies survive ethanol-rearing compared with 68% of controls (Supporting Information, Figure S1). Finally, heterozygosity for the lethal null allele *Cat^n1^* reduces viability in ethanol to 48%, which, while not statistically significant, nonetheless shows the same trend (Figure S1)

We obtained similar results when we reared flies mutant for the “antioxidant genes” *Glutathione Synthase* (*GS*), *Peroxidase* (*Pxd*), and *rosy* (*ry*) on ethanol-containing food. Glutathione synthase catalyzes the production of glutathione, a powerful antioxidant, from γ-glutamylcystiene and glycine, whereas Peroxidase converts hydrogen peroxide to water and molecular oxygen, oxidizing glutathione to glutathione disulfide in the process. *ry* encodes xanthine dehydrogenase, which converts xanthine to the antioxidant molecule urate. All three of these mutant strains were exquisitely sensitive to developmental ethanol exposure: to avoid a “floor effect” in which we recovered fewer than 10% of our mutant flies, it was necessary to reduce the ethanol concentration in the food to 3%, which has little to no effect on control animal survival. At this concentration, half of *GS* and *Pxd* mutant flies survived to eclosion, and only 29% of the urate-null *ry^506^* flies survived (Figure 3C).

Interestingly, flies mutant for *urate oxidase* (*uro*), which catalyzes the conversion of urate to allantoin, were also sensitive to ethanol-induced developmental lethality, showing only 22% survival in 3% ethanol (Figure 3C). This is an unexpected result because quantitative RT-PCR (qRT-PCR) demonstrates that the *uro* mutation is a very strong loss-of-function mutation (we could detect no transcript in the mutant line, not shown), and the expected effect of mutating *uro* would be a build-up of urate, the opposite of the effect the *ry* mutation. In addition, while ry, GS, and Pxd have no effect on development time in ethanol, mutation of *uro* leads to suppression of the developmental delay caused by ethanol, exactly as predicted and in opposition to its effect on survival (Figure 3D). At present, these results are unexplained. However, the majority of our data indicate that ethanol exerts its effects on survival through an increase in the production of reactive oxygen species (ROS).

### Ethanol exposure leads to changes in antioxidant gene expression

Ethanol exposure is known to lead to increases in oxidative stress in at least two ways: through the increased production of free radicals and through decreased expression of antioxidant genes, including Cu/Zn superoxide dismutase (Cu/Zn SOD) (Polavarapu *et al.* 1998; Halliwell 1999). In addition, expression of antioxidant genes often increases as a result of the increased production of ROS (Yu 1994; Halliwell 1999; Wu and Cederbaum 2003). We used qRT-PCR to examine the expression of *GS*, *Cat*, *Cu/Zn SOD*, *uro*, and both *Pxd* transcripts (*Pxd-RA* and *Pxd-RB*) in third-instar larvae developing in ethanol. Of these, *GS*, *Cat*, and *Pxd-RB* showed dramatic changes in transcription (Figure 3E). The expression of *Pxd-RB* was reduced 12-fold in ethanol-reared larvae, whereas the expression of *Pxd-RA* was reduced two-fold. These changes would be expected to result in an increase in hydrogen peroxide and peroxide radicals.

By contrast with *Pxd*, *Cat* was *upregulated* five-fold in ethanol-exposed larvae, which is likely a response to increased cellular production of ROS on ethanol exposure. Similarly, *GS* showed a 12-fold upregulation in expression, which is consistent with the increased cellular production of glutathione that would be expected in response to increased peroxide production. Finally, *SOD* showed a modest but consistent increase in expression (1.7-fold) in ethanol-reared larvae. The expression of *uro* was not substantially changed.

In addition to the gene expression changes described above, microarray analysis revealed that a cluster of genes involved in the detoxification of ROS are differentially expressed in ethanol-reared larvae (Table 2). These include two glutathione-S-transferase (GST)-encoding genes (*GstD4* and *GstD8*) that are downregulated four-fold as a result of ethanol exposure, as well as three genes encoding cytochrome P450 enzymes (Cyp6a2, Cyp4d21, and Cyp4e3). GSTs catalyze the conjugation of glutathione to xenobiotic substrates as a first step in detoxification, and their loss would be predicted to increase sensitivity to ethanol-induced ROS toxicity. Cyp450 enzymes catalyze a wide variety of oxidation reactions, many involved in xenobiotic detoxification. These reactions generate ROS. Cyp4e3, in particular, was induced 83-fold in response to ethanol, a result we have confirmed with qRT-PCR (121-fold increase). Finally, three genes encoding pantetheine hydrolases are upregulated 4-fold to 12-fold in ethanol-reared larvae. Pantetheine hydrolases, or vanins, catalyze the conversion of pantetheine to pantothenic acid (vitamin B5) and 2-aminoethanethiol. In mice, *vanin-1* expression is induced by oxidative stress, and mutation of *vanin-1* confers a glutathione-mediated resistance to oxidative stress (Berruyer *et al.* 2004).

**Table 2:** Genes involved in lipid metabolism are overrepresented among transcripts altered by larval ethanol exposure

Gene Ontology Term	Total on Array	Number Changed	Percent Changed	Z Score	*P*
Phospholipase A1 activity	11	6	55%	10.1	0.0
Pantetheine hydrolase activity	3	3	100%	8.5	0.0
Glucosylceramidase activity	3	3	100%	9.9	0.0005
Triglyceride lipase activity	38	8	21%	6.6	0.002
Sphingolipid metabolic process	22	4	18%	4.2	0.036

Analysis using the GO Elite software package indicates that five categories of lipid metabolism genes are significantly overrepresented among the transcripts altered by at least four-fold in ethanol-reared third instar larvae. The Z score is a normal approximation to the hypergeometric distribution. *P* values were calculated using Fisher’s exact test.

Our gene expression data indicate that larvae exposed to ethanol undergo a complex set of gene expression changes, some of which are indicative of an organismal response to increased production of ROS (upregulation of *Cat*, *GS*, and *Sod*), whereas other changes would be expected to contribute to the overall increase in sensitivity to oxidative stress by reducing the fly’s ability to respond to the increased stress (*Pxd*, *GstD4*, and *GstD8*).

### Larval ethanol exposure disrupts fatty acid metabolism and leads to lipid accumulation

Ethanol may increase the production of ROS in a number of ways, including increased activity of the respiratory chain, stimulation of Cyp450s, which increase ROS production, reduction in the expression or activity of antioxidant molecules (especially GSH), and direct production of an ethanol-derived radical (the 1–hydroxyethyl radical) (Wu and Cederbaum 2003). As described above, we have evidence for at least two of these mechanisms (Cyp450 activity and altered antioxidant gene expression). In addition, we have compelling evidence that, in our system, ethanol is inducing oxidative stress through disruption of long-chain fatty acid metabolism.

We performed two screens designed to identify ethanol’s molecular targets during development. The first of these was a microarray screen comparing mRNA isolated from ethanol-reared third instar larvae to those of controls. This screen would be expected to identify genes that are transcriptional targets of ethanol exposure. The complete results of this screen are provided in Table S1 and Table S2. The second screen was a forward genetic screen for *P* element–induced mutations that alter survival or development time of flies growing in 7% ethanol. This screen would be expected to identify some ethanol target genes but, more importantly, genetic and biochemical pathways that are important for the developmental effects of ethanol, whether they are direct targets. The full results of the genetic screen will be presented elsewhere. Both screens yielded results that strongly suggest defective long chain fatty acid metabolism as a cause of ethanol-induced oxidative stress.

Our microarray analysis revealed changes in the expression of at least 24 transcripts whose products are directly involved in fatty acid metabolism or synthesis, and most of these changes would be predicted to result in accumulation of fatty acids. We performed an overrepresentation analysis using the GO Elite software package, which revealed that five gene ontology (GO) terms associated with lipid metabolism were statistically overrepresented among the transcripts whose expression was altered at least four-fold by DAE (Table 2). There are 77 transcripts identified by these five GO terms on the array, and 24 (31%) show changes on DAE. By comparison, for the entire dataset (18,952 transcripts on the array), 1666 (9.8%) were changed at least four-fold by DAE (Table S1 and Table S2)

These genes include eight genes encoding triglyceride lipases, all of which are reduced by at least five-fold in ethanol-reared larvae and, in one case, by more than 200-fold (Table 3). The two genes demonstrating the greatest downregulation in ethanol-reared larvae both encode triglyceride lipases (CG6277, 216-fold; CG6283, 163-fold), and three of the top 11 most downregulated genes fall into this cluster.

**Table 3:** Larval gene expression changes in response to ethanol

	Gene	Expression Change	Protein Function	Predicted Effect
Oxidative stress-related	Cyp4e3	121-fold increase	Cytochrome P450	Increased ROS
	Cyp6a2	4.2-fold increase	Cytochrome P450	Increased ROS
	CG32750, CG32751, vanin-like	3.6- to 12.5-fold increases	Pantetheine hydrolases	Increased sensitivity to ROS/increased fatty acid synthesis
	Cyp4d21	6.7-fold decrease	Cytochrome P450	
	GSTD8	4.6-fold decrease	Glutathione-S-transferase	Increased ROS
	GSTD4	4.3-fold decrease	Glutathione-S-transferase	Increased ROS
Lipid metabolism				
	Gpdh	4.1-fold increase	Glycerol-3-phosphate dehydrogenase	Fatty acid accumulation
	GlcT-1	4-fold increase	Ceramide glucosyltransferase	Fatty acid accumulation
	Acox57D-d	3.9-fold increase	Acyl-coenzyme A oxidase	Fatty acid accumulation
	Gpo-1	3.2-fold increase	Mitochondrial glycerol-3-phosphate dehydrogenase	Fatty acid accumulation
	CG17191, CG17192, CG6271, CG6277, CG6283, CG6295, CG8093	5.1- to 216-fold decreases	Triglyceride lipases	Fatty acid accumulation
	CG31148, CG31414, CG33090	6.4- to 11-fold decreases	Glucosylceramidases	Fatty acid accumulation
	aSMase	9.9-fold decrease	Acid Sphingomyelinase	Reduced ceramide
	ATPCL	8.3-fold decrease	ATP citrate lyase	Possible increased storage of triglycerides

RNA was isolated from third instar wandering larvae reared in either 0% or 7% ethanol, and gene expression was compared using Affymetrix Drosophila 2.0 microarray chips. Data are presented as fold-change in 7% reared larvae relative to control larvae (developing in 0% ethanol).

In addition to lipases, we identified a cluster of downregulated genes encoding glucosylceramidases (Table 3). Glucosylceramidases catalyze the release of D-glucose and N-acylsphingosine, a free ceramide, from D-glucosyl-N-acylsphingosine. In mammals, glucosylceramidase deficiency leads to lipid accumulation and is the cause of the human neurological disorder Gaucher disease. Two additional genes (GlcT-1 and aSMase) are altered in ways that would be similarly predicted to decrease free ceramide levels. Table 2 details the complete list of fatty acid metabolism genes with altered expression in ethanol-reared larvae.

Finally, it is worth noting that pantothenic acid is required for synthesis of coenzyme A; thus, increased fatty acid biosynthesis and accumulation are among the potential effects of increased pantotheine hydrolase activity.

**Figure 4:**
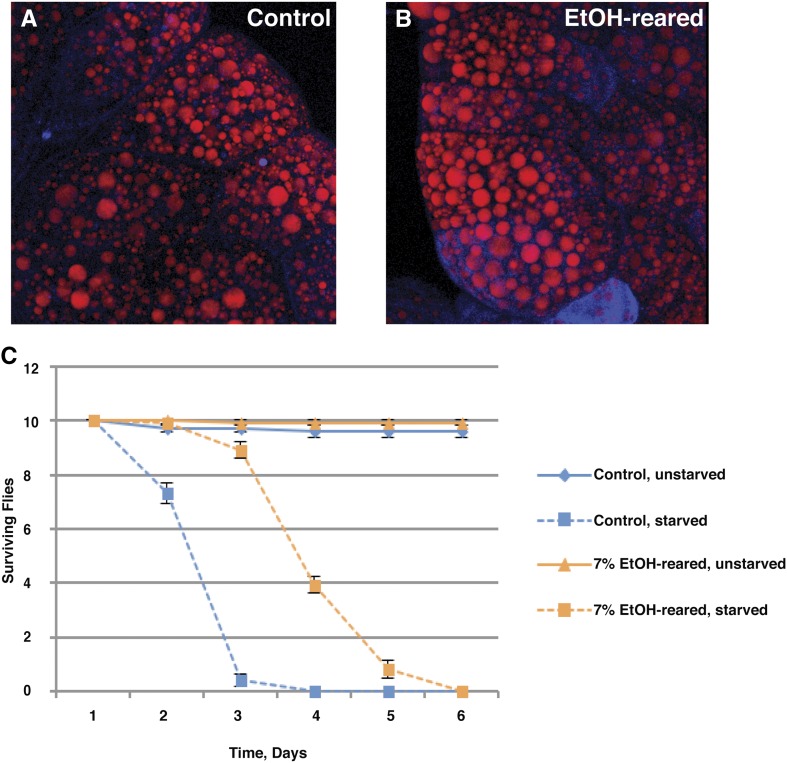
Ethanol-reared flies show increased lipid storage and starvation resistance. (A) Confocal reconstruction of a Nile Red–stained fat body dissected from a wandering third instar larva that was reared in control food containing no ethanol. (B) Confocal reconstruction of a Nile Red–stained fat body dissected from an ethanol-reared wandering third instar larva. Note the increased diameter of fat droplets relative to the control fat body shown in (A). (C) Adult flies reared in ethanol show increased resistance to starvation, with median survival of an additional 1.5 d on 1% agarose medium, which provides water but no nutrients. Median survival of control flies: 2.3 d; median survival of ethanol-reared flies: 3.8 d; *P* < 0.0001, Student’s t test, N = 10.

The gene expression changes described above and in Table 2 and Table 3 would be expected to result in lipid accumulation. To test this prediction, we examined the lipid content of ethanol-reared larvae. Nile Red staining of the larval fat body shows an increase in lipid storage, as lipid droplets are increased in both number and size (Figure 4, A and B). In addition, ethanol-reared flies are significantly resistant to starvation. We transferred 0- to 2-d-old adult flies to vials containing 1% agarose, which provides a source of water but no nutrition, and compared survival between ethanol-reared flies and control flies. Ethanol-reared flies lived longer, with a median survival time of 3.8 d compared with 2.3 d for control flies (Figure 4C). This increase is most likely due to the observed increase in fat storage in ethanol-reared flies.

### Ethanol induces oxidative stress by disrupting long-chain fatty acid metabolism

**Figure 5:**
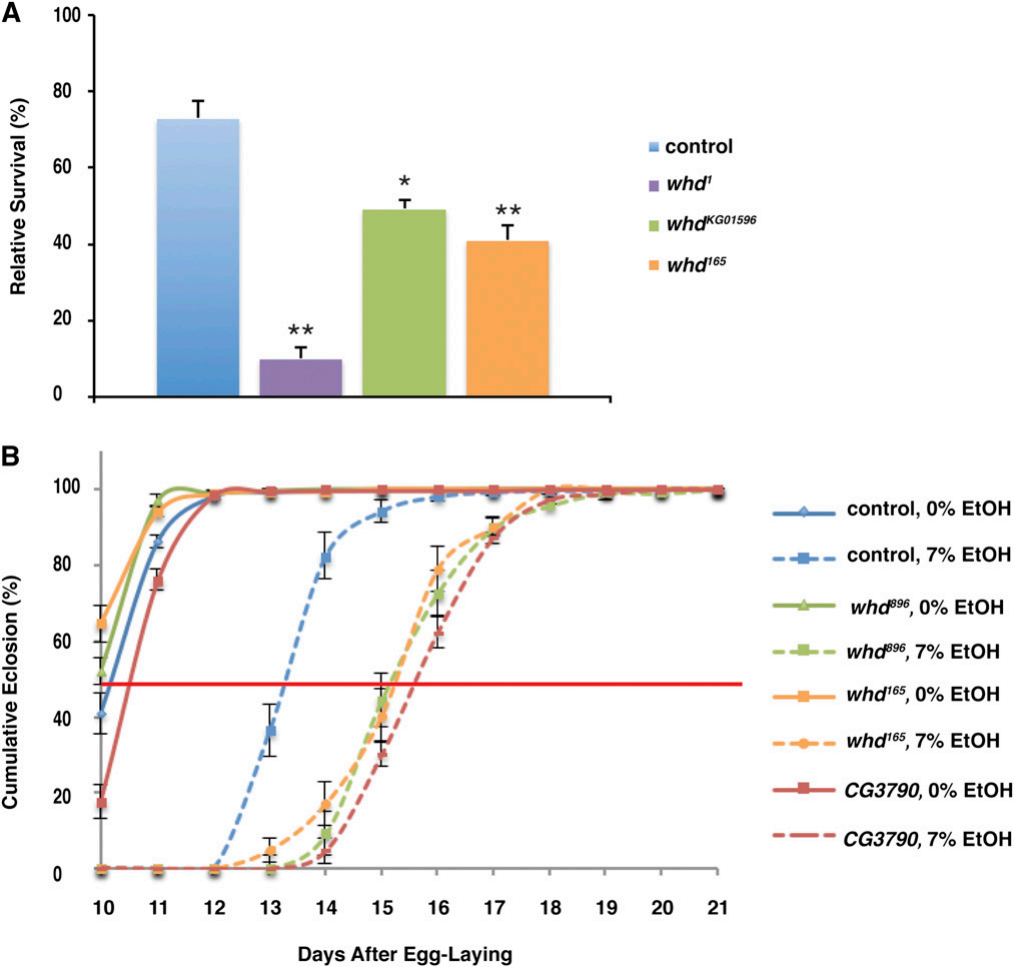
whd and CG3790 mutants are sensitive to developmental ethanol effects. (A) *whd* mutant flies are sensitive to ethanol-induced developmental lethality. Survival has been normalized to age- and genotype-matched controls reared in ethanol-free food. N = 4. *P < 0.05, **P < 0.01, one-way ANOVA with Tukey HSD posthoc analysis. (B) *whd^165^*, *whd^896^*, and *CG3790* mutant flies are sensitive to ethanol-induced developmental delay, taking approximately 2 additional days to reach 50% eclosion in ethanol-containing medium. Time to 50% eclosion was: WT 0% EtOH, 10.2 ± 0.1 d; WT 7% EtOH, 13.3 ± 0.14 d (*P* < 0.01, one-way ANOVA with Tukey HSD posthoc analysis); *whd^896^* 0% EtOH, 10.0 ± 0.07 d; *whd^896^* 7% EtOH, 15.4 d ± 0.14 d (*P* < 0.01 *vs.* both 0% controls and WT 7% EtOH-reared, one-way ANOVA with Tukey HSD post hoc analysis); *whd^165^* 0% EtOH, 9.8 ± 0.08 d; *whd^165^* 7% EtOH, 15.4 d ± 0.09 d (*P* < 0.01 *vs.* both 0% controls and WT 7% EtOH-reared, one-way ANOVA with Tukey HSD post hoc analysis); *CG3790* 0% EtOH, 10.6 ± 0.05 d; *CG3790* 7% EtOH, 15.8 d ± 0.09 d (*P* < 0.01 *vs.* both 0% controls and WT 7% EtOH-reared, one-way ANOVA with Tukey HSD post hoc analysis). Data are normalized to the total number of flies that eclosed for that treatment condition and genotype.

We performed a genetic screen for mutations that alter survival or development time of flies growing in 7% ethanol-containing medium. Among the mutations we recovered were two alleles of *withered* (*whd*). We have designated these alleles *whd^165^* and *whd^896^*. Both of these alleles resulted in increased developmental delay when reared in ethanol (Figure 5B), and *whd^165^* is also sensitive to the developmental lethality resulting from ethanol exposure (Figure 5A). We subsequently tested two additional alleles of *whd*, *whd^1^*, and *whd^KG01596^*. Both of these alleles showed increased ethanol-induced developmental mortality (Figure 5A). In particular, *whd^1^*, which is a null allele (Strub *et al.* 2008) and exhibits only 43% survival on control food relative to wild-type flies, was also profoundly sensitive to ethanol, with only 10% of *whd^1^* flies surviving to adulthood when reared in ethanol (compared with the same genotype reared on control food).

*whd* encodes the *Drosophila* homolog of carnitine parmitoyltransferase I (CPTI) (Strub *et al.* 2008). CPTI is located in the outer mitochondrial membrane and catalyzes the transfer of the acyl groups of long-chain fatty acyl-CoA to carnitine. The resulting acyl-carnitine can then be transported through the outer and inner mitochondrial membranes to the matrix. Thus, CPTI is an essential enzyme for β-oxidation of long-chain fatty acids. In mammals, CPTI deficiency leads to elevation of free fatty acid levels due to the shunting of long-chain fatty acids away from the mitochondria (Rasmussen *et al.* 2002). In *Drosophila*, *whd* mutants are also sensitive to oxidative stress induced by paraquat and heavy metals (Strub *et al.* 2008). Thus, our identification of two ethanol-sensitive alleles of *whd* supports our hypothesis that developmental ethanol exposure causes oxidative stress by disrupting fatty acid metabolism.

In addition to *whd*, we recovered an allele of *CG3790*. *CG3790* is predicted to encode the *Drosophila* carnitine transporter (CT), which catalyzes the movement of acyl-carnitine across the inner mitochondrial membrane. This allele, like both alleles of *whd*, results in an increased developmental delay when reared in ethanol (Figure 5B). Our data demonstrate that ethanol exposure causes disruptions in lipid metabolism as well as oxidative stress, and that mutations disrupting long-chain fatty acid metabolism confer sensitivity to ethanol-induced oxidative stress.

## Discussion

DAE causes a wide array of deleterious effects, including low birth weight, increased fetal mortality, developmental delays, craniofacial dysmorphologies, neurobehavioral abnormalities, and moderate to severe intellectual disabilities (Hanson *et al.* 1976; Streissguth *et al.* 1980, 2004; Colangelo and Jones 1982; Kodituwakku 2007). Thus, although it is known that DAE is a powerful teratogen, the mechanisms underlying the myriad of potential phenotypes are not well-understood. This is, in part, due to the fact that the model systems that best simulate the effects of DAE in humans (vertebrate systems including mice and *Xenopus*) (Allan *et al.* 2003; Yelin *et al.* 2005) are generally not amenable to forward genetic analysis, making unbiased identification of ethanol’s targets difficult.

We have established a *Drosophila* model of DAE to begin to uncover additional developmental targets of ethanol. Here, we show that the lethality and developmental delay caused by ethanol exposure are largely due to oxidative stress. Mutants with reduced ability to cope with oxidative stress were similarly sensitive to ethanol-induced lethality and developmental delay, whereas experimentally increasing antioxidant gene activity conferred resistance to these effects. In addition, hydrogen peroxide and ethanol act synergistically, indicating a common target pathway. Finally, we see a widespread pattern of gene expression changes consistent with increased oxidative stress. Ethanol appears to increase oxidative stress through several mechanisms, and we present data for a novel mechanism in which ethanol exposure leads to increased levels of ROS through disruption of lipid metabolism during larval development.

### Multiple mechanisms for ethanol-induced oxidative stress

Ethanol can increase oxidative stress by increasing the production of ROS, or by reducing cellular antioxidant protections. The metabolism of ethanol to acetaldehyde leads to the production of two molecules of NADH, which leads to a concomitant increase in activity of the respiratory chain, increased use of O_2_, and therefore increased production of ROS. In addition, acetaldehyde itself interacts with proteins and lipids in ways that lead to the production of free radicals (Wu and Cederbaum 2003). Ethanol also stimulates the activity of Cyp450 detoxification proteins. Stimulation of Cyp450 proteins leads to oxidation of the xenobiotic compound (in this case, ethanol or its metabolites), and the further metabolism of these intermediates generates reactive oxygen species. Finally, ethanol exposure can result in the production of the 1-hydroxyethlyl radical.

In addition to the direct increase in ROS, alcohol exposure handicaps the cell’s ability to reduce oxidative stress. Alcohol also reduces the expression of antioxidant molecules, decreasing the cell’s ability to combat oxidative stress and leading to an increase in ROS production. Ethanol exposure can cause reduced expression and activity of SOD in liver tissue (Polavarapu *et al.* 1998; Halliwell 1999). In addition, alcohol depletes GSH levels in the liver, presumably by reducing the expression or activity of the enzymes needed for GSH production (Wu and Cederbaum 2003). Ethanol also interferes with the transport of GSH into the mitochondria, increasing the depletion of mitochondrial GSH (Fernández-Checa *et al.* 1997).

We have evidence for at least two of these mechanisms. We show upregulation of two Cyp450 proteins (Table 2), the activation of which would be expected to lead to increased production of ROS. In addition, we observed upregulation of Cat, GS, and Sod, indicating a cellular response to higher levels of ROS. Simultaneously, we have evidence of ethanol-induced downregulation of antioxidant genes: the expression of *Pxd*, *GstD4*, and *GstD8* are all reduced in ethanol-reared larvae.

Interestingly, although our data indicate that both developmental delay and mortality are exacerbated by oxidative stress, not all manipulations affect both phenotypes in the same way. Mutations in *Pdk1* enhance the developmental delay caused by ethanol while simultaneously rescuing the lethality. Similarly, mutation of *uro* rescues the developmental delay while enhancing ethanol-induced lethality. Some alleles of *whd* affect both delay and lethality, whereas others affect only lethality. Finally, mutations in *ry*, *GS*, and *Pxd* affect only lethality and not delay. This indicates that the two phenotypes are mechanistically separable, which is not a surprise given that we have previously shown them to be separable in terms of the period of developmental sensitivity for each. It is possible, given the variety of ways in which ethanol can induce oxidative stress, that developmental delay and lethality are due to different “types” of oxidative stress, or that different mechanisms predominate depending on developmental stage and/or tissue type. At present, however, we have no direct evidence for this hypothesis.

Finally, although mutation of *ry* and *uro* should have opposite effects on the production and accumulation of urate in cells, we found that ry^506^ and *uro^f04888^* resulted in the same phenotype: sensitivity to ethanol-induced lethality (Figure 3C). One explanation for these results may lie in the complex effects of urate on oxidative stress. In mammalian cells, urate can have either antioxidant or pro-oxidant effects, depending on cellular and chemical context. Most relevant to this work, urate exerts a pro-oxidant effect hydrophobic environments; in adipocytes, urate uptake triggers increased ROS production as a result of activation of NADPH oxidase (Sautin *et al.* 2007). Thus, the sensitivity of *uro^f04888^* flies to DAE may be a result of increased production of ROS on urate build-up in the larval fat body.

Similarly, DAE results in complex changes in antioxidant gene expression, as seen in Figure 3E. Some genes, including *GS* and *Cat*, are upregulated as a result of DAE, whereas *Pxd* expression is reduced. These differences are most likely due to the different mechanisms by which ethanol induces oxidative stress. Reduced expression of antioxidant molecules (such as Peroxidase) by ethanol, as well as the direct production of free radicals, would be expected to induce expression of other gene products as a response to the increase in oxidative stress. Thus, we hypothesize that when *Pxd* expression is reduced on DAE, part of the cellular response is to increase expression of *GS* and *Cat*.

### Ethanol-induced perturbations of lipid metabolism

Ethanol-exposed larvae have increased circulating triglyceride levels as well as increased lipid storage, as demonstrated by the increased size of the larval fat body. We have previously shown that Dilp signaling is reduced 25–50% in ethanol-reared larvae (McClure *et al.* 2011), and the obesity phenotype observed in our larvae is almost certainly due to reduced Dilp expression. Adult *Drosophila* with ablated insulin-producing cells (IPCs) also display increased circulating and stored lipids (Hwangbo *et al.* 2004; Broughton *et al.* 2005; Pospisilik *et al.* 2010; Varghese *et al.* 2010).

Fatty acid accumulation causes oxidative stress and cellular toxicity, and can lead to apoptosis (Listenberger *et al.* 2001, 2003). Similarly, obesity in mammals is associated with oxidative stress (Furukawa *et al.* 2004; Fernández-Sánchez *et al.* 2011). We found that mutations in *whd*, the *Drosophila* homolog of CPTI (Strub *et al.* 2008), are sensitive to ethanol-induced developmental toxicity. CPTI is located in the outer mitochondrial membrane and catalyzes the transfer of the acyl groups of long-chain fatty acyl-CoA to carnitine. The resulting acyl-carnitine can then be transported through the outer and inner mitochondrial membranes to the matrix. In mammals, CPTI deficiency leads to elevation of free fatty acid levels due to the shunting of long chain fatty acids away from the mitochondria (McGarry *et al.* 1983; Rasmussen *et al.* 2002). In addition, a mutation in the *Drosophila* carnitine transporter (CT) is also sensitive to larval ethanol exposure. Taken together, our data strongly suggest that, in addition to the two mechanisms described above, ethanol causes oxidative stress during development by disrupting fatty acid metabolism. We are currently conducting a series of experiments to manipulate the levels and types of fatty acids present in the larval diet and measure the effects of these changes on ethanol-induced oxidative stress to explicitly connect the lipid metabolism defect with increased production of ROS and to test dietary changes as a potential treatment for some of the deleterious effects of DAE.

The finding that lipid metabolism is disrupted by DAE is intriguing for two reasons. First, to our knowledge, very little is known about lipid metabolism and storage in other models of FASD. We were able to find a single report of a model of FASD in guinea pigs examining DHA accumulation during gestation (Skinner 2002). This reference did not discuss lipid accumulation as a cause of fetal alcohol effects.

Second, lipid accumulation is known to result in neuronal death during development. Tay-Sachs disease, a developmental neurodegenerative disorder, is caused by a mutation in the lysosomal protein hexosaminidase A. This mutation leads to the build-up of the sphingolipid GM2 ganglioside in the brain (Mahuran 1999). Many nervous system disorders, including Alzheimer disease, Parkinson’s disease, Neiman-Pick disease (NPD), and schizophrenia, are associated with dysregulation of lipid metabolism, often in conjunction with oxidative stress (Adibhatla and Hatcher 2007).

Of particular interest for this work is NPD. All three forms of NPD are associated with severely reduced levels of acid Sphingomyelase (aSMase), which results in lysosomal accumulation of sphingolipids. In mouse models, replacement of aSMase activity was effective as a treatment for type B NPD, whereas inhibition of glycosphingolipid synthesis prolonged life in a mouse model of type C NPD (Miranda *et al.* 2000; Mukherjee and Maxfield 2004; Vance 2006). We have identified a mutation in the gene *Npc1a*, one of the *Drosophila* homologs of the NPD type C gene, which is exquisitely sensitive to ethanol exposure during development (T. Logan-Garbisch, C. Wu, and R. L. French, unpublished results). In addition, aSMase is reduced 10-fold in ethanol-reared larvae, which is comparable with the levels found in individuals with NPD type B. Finally, we have shown downregulation of three glucosylceramidases (Table 2), which would be predicted to result in a build-up of glucosylsphingolipids. These data make sphingolipid accumulation an attractive target for some of the deleterious effects of ethanol exposure in cases of FASD.

## Supplementary Material

Supporting Information
