# Supplementary material for: Developmental Ethanol Exposure Leads to Dysregulation of Lipid Metabolism and Oxidative Stress in Drosophila
Source: G3 (Bethesda). 2014 Nov 11;5(1):49–59. doi: 10.1534/g3.114.015040 (PMC4291469; doi:10.1534/g3.114.015040)
Supplement: Supporting Information [file supp_5_1_49__index.html]

Developmental Ethanol Exposure Leads to Dysregulation of Lipid Metabolism and Oxidative Stress in Drosophila — Supporting Information 

# Developmental Ethanol Exposure Leads to Dysregulation of Lipid Metabolism and Oxidative Stress in *Drosophila*

## Supporting Information for Logan-Garbisch *et al.*, 2015

**Files in this Data Supplement:**

- Figure S1 - Additional alleles of *Cat* are sensitive to ethanol. (PDF, 146 KB)
- File S1 - Dataset (.xlsx, 157 KB)
- Table S1 - Complete results of the microarray analysis. Data are presented as the ratio of signal from RNA isolated from control 3rd-instar larvae to that from 3rd-instar larvae reared in 7% ethanol. (.xlsx, 958 KB)
- Table S2 - Transcripts displaying at least a four-fold change in expression after microarray analysis. Data are presented as the ratio of signal from RNA isolated from control 3rd-instar larvae to that from 3rd-instar larvae reared in 7% ethanol. (.xlsx, 117 KB)
