# Supplementary material for: Developmental Ethanol Exposure Leads to Dysregulation of Lipid Metabolism and Oxidative Stress in Drosophila
Source: G3 (Bethesda). 2014 Nov 11;5(1):49–59. doi: 10.1534/g3.114.015040 (PMC4291469; doi:10.1534/g3.114.015040)
Supplement: Supporting Information [file supp_g3.114.015040_FigureS1.pdf]

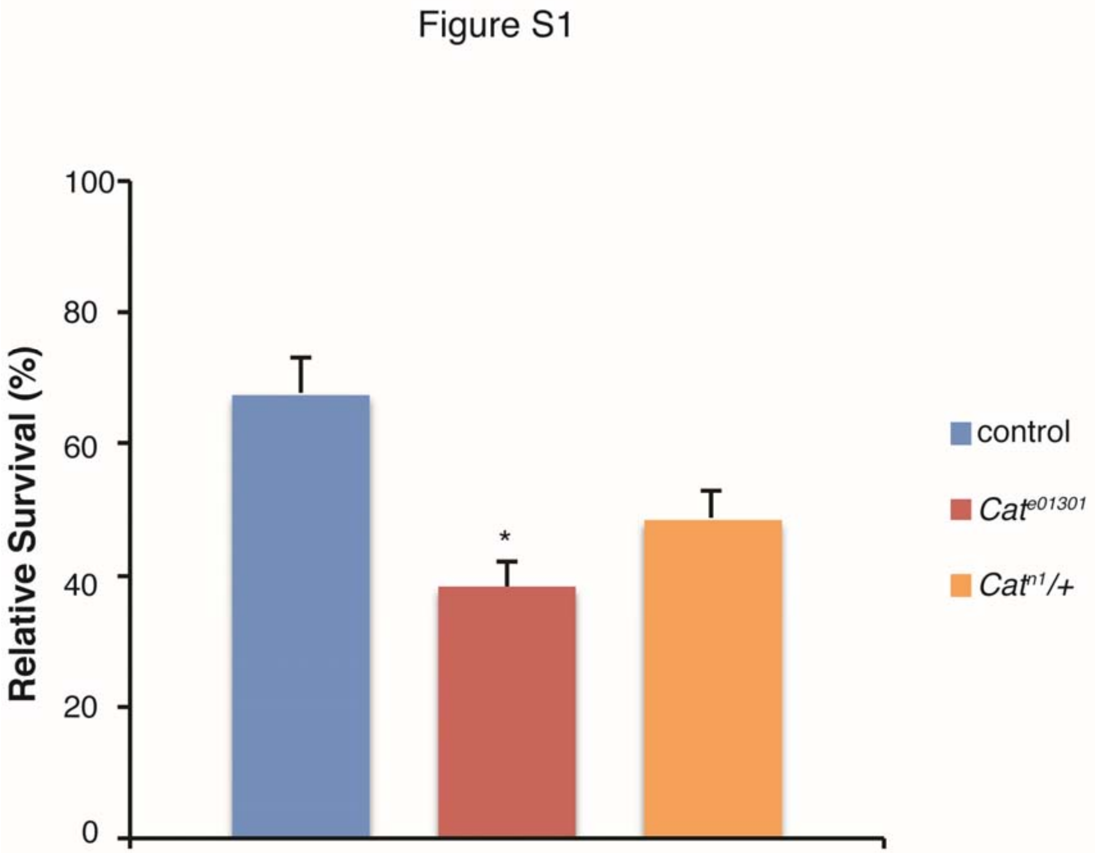

Figure S1 Additional alleles of *Cat* are sensitive to ethanol. Flies homozygous for *Cat*^{e01301} are sensitive to ethanol-induced developmental lethality, and flies heterozygous for the null allele *Cat*^{n1} show a trend towards increased lethality, but this effect does not achieve statistical significance. *: $P < 0.05$, one-way ANOVA with Tukey HSD posthoc analysis.
